# Influence of race/ethnicity and income on the link between adverse childhood experiences and child flourishing

**DOI:** 10.1038/s41390-020-01188-6

**Published:** 2020-10-12

**Authors:** Ellen Goldstein, James Topitzes, Julie Miller-Cribbs, Roger L. Brown

**Affiliations:** 1 Madison Department of Family Medicine and Community Health, University of Wisconsin, Madison, WI USA; 2 Milwaukee Helen Bader School of Social Welfare and Institute for Child and Family Well-Being, University of Wisconsin, Madison, WI USA; 3 Anne and Henry Zarrow School of Social Work, University of Oklahoma-Tulsa, Tulsa, OK USA; 4 Design and Statistics Unit, Madison Schools of Nursing and School of Medicine and Public Health, University of Wisconsin, Madison, WI USA

## Abstract

**Background:**

The impact of early adversity increases the risk of poor outcomes across the life course. Identifying factors that protect against or contribute to deleterious life outcomes represents an important step in resilience promotion among children exposed to adversity. Informed by resilience science, we hypothesized that family resilience mediates the relationship between adverse childhood experiences (ACEs) and child flourishing, and these pathways vary by race/ethnicity and income.

**Methods:**

We conducted a secondary data analysis using the 2016–17 National Survey of Children’s Health data reported by parents/guardians for 44,686 children age 6–17 years. A moderated-mediation model estimated direct, indirect, and total effects using a probit link function and stacked group approach with weighted least square parameter estimates.

**Results:**

The main variables were related in expected directions. Family resilience partially mediated the ACEs-flourishing association. Although White and socioeconomically advantaged families were more likely to maintain family resilience, their children functioned more poorly at high-risk levels relative to Black and Hispanic children and across income groups.

**Conclusion:**

Children suffer from cumulative adversity across race/ethnicity and income. Partial mediation of family resilience indicates that additional protective factors are needed to develop comprehensive strategies, while racial/ethnic differences underscore the importance of prevention and intervention programs that are culturally sensitive.

**Impact:**

- The key message of the article reinforces the notion that children suffer from cumulative adversity across race/ethnicity and income, and prevention of ACEs should be the number one charge of public policy, programs, and healthcare.
- This is the first study to examine family resilience in the National Survey Children’s Health (NSCH) data set as mediating ACEs-flourishing by race/ethnicity and family poverty level.
- Examining an ACEs dose–response effect using population-based data within the context of risk and protective factors can inform a public health response resulting in a greater impact on prevention efforts.

## Introduction

Adverse childhood experiences (ACEs) are common and associated with negative health and social outcomes.^1^ Nearly half of children in the U.S. have experienced at least one childhood adversity prior to age 18 with a much higher prevalence among children of color and socioeconomically vulnerable children.^2^ Early life adversity can undermine learning and is linked to a range of school-related problems,^3^ and can jeopardize psychological health and well-being.^4^ According to resilience science, identifying factors and processes that influence development represents an important step in the promotion of individual and family resilience.^5^ The role of family-level variables is central to promoting child flourishing and counteracting adversity across the life course. Using a nationally representative data set, this study examines the relationship between cumulative ACEs and flourishing outcomes mediated by family resilience and moderated by race/ethnicity and family poverty level (FPL) in children ages 6–17 years.

### Adverse childhood experiences

ACEs, often chronic and intergenerational, are broadly characterized as abuse, neglect, and household dysfunction prior to age 18 years.^6^ In recent years, the ACEs framework has expanded beyond the household-level to encompass community-level stressors such as economic hardship, discrimination, witnessing neighborhood violence, and bullying.^7^ Experiencing mistreatment of any kind and growing up in an unpredictable environment in which safety, stability, and trusting and nourishing relationships are disrupted can impact neurobiological, cognitive, and social-emotional development.^8^ Cumulative ACEs are predictive of cascading life course problems such as academic failure, problems with socio-emotional maladjustment, and poor health outcomes.^1,9^ Evidence continues to mount that cumulative ACEs exposures, rather than individual ACE experiences, yield the most negative impacts on overall well-being.^7^ Research suggests that the cumulative effect of stressors in families may lead to deteriorating family resilience as well as worsening of child and adolescent outcomes.^10,11^

### ACEs and child flourishing

Flourishing relates to well-being and for this study is a parent-reported measure based on indicators of whether children show interest and curiosity in learning new things, can focus and persist to achieve goals and are able to regulate emotions and behaviors in challenging situations.^12^ Children who have experienced at least one ACE are significantly less likely to flourish compared to those with no ACEs.^13^ Children exposed to adversity are more susceptible to toxic stress and may have an increased vulnerability to stress reactivity.^8^ Chronic stress has negative effects on the brain and body, which can lead to concomitant difficulties with learning and memory, executive functioning, and emotional regulation in the absence of protective factors and processes. Innate abilities to engage with others, process information, learn new lessons, and maintain flexibility or resilience diminish as the survival response takes over from these secondary social processes.^14^ Yet children somehow manage to flourish despite the challenge of having experienced ACEs and can learn healthier ways of responding to adverse events. For example, children who demonstrate resilience or the ability to stay calm and in control when faced with a challenge have higher rates of school engagement.^15^

### Resilience and protective factors

Evidence suggests that, despite adverse conditions, the ability to demonstrate resilience persists.^16^ The difference between those who adapt and do well and those who do not maintain or re-establish baseline functioning after experiencing adversity is the presence of protective factors such as strong social support from friends, family, and community.^17^ Research on resilience indicates that healthy relationships protect against adversity and strengthen the ability to effectively manage stress and cope with adversity.^5^ Protective factors operate at the community, family, and individual levels and can contribute to well-being in children as measured by school engagement and achievement, social adjustment and behavior, and mental and physical health.^18–20^ For the current study, analyses include family-level factors as potential mechanisms that contribute to child resilience.

### Importance of family relationships

The importance of safe, stable, nurturing relationships across development contributes to the health and well-being of individuals across the lifespan.^7^ Family resilience refers to the ability of a family to withstand and rebound from disruptive life events. Family processes can support and protect the biological, psychological, and social functioning of children, whose development is most vulnerable to adversity.^21^ Moreover, supportive relationships and positive family functioning can buffer the impact of stress. The capacity to process, integrate and make sense of traumatic experiences happens through healthy relationships that provide emotional support.^22^ Good caregivers have the capacity to offer safety, protection, and reassurance under conditions of stress, which provide an important basis for emotional regulation and healthy attachments when repeated over time.

### Racial/ethnic and socioeconomic disparities

Although ACEs affect people across racial/ethnic and socioeconomic divides, patterns of adversity are differentially distributed across race/ethnicity and income with some communities facing a disproportionate burden of trauma as compared to others.^4^ These inequities are most evident in families of color, those living in poverty, and in community contexts with vastly different resources.^23,24^ Researchers have explored the interconnected and sometimes overlapping system of disadvantage that commonly occurs within the context of adverse community environments.^25–27^ Certain groups are at higher risk for ACE exposure including those who are poor, reside in unsafe neighborhoods, lack access to health care, or are enrolled in public insurance programs.^15^ Concentrated disadvantage exists among Black and Hispanic youth living in urban areas, who are exposed at a higher rate to traumatic events than their white peers.^28,29^ As a result, children and families of color residing in low-income neighborhoods are particularly vulnerable to the impact of ACEs on their mental and physical health and socio-emotional learning.^24^

### Present study objectives

While extant literature has focused strongly on the long-term, negative health consequences of ACEs, less is known about the factors and processes that may offer protection against the corrosive health effects of early life trauma and socioeconomic disadvantage. Building on previous population-based studies, the current study estimates the direct effect relationships of cumulative ACEs and family resilience on three child flourishing indicators using the 2016–2017 NSCH combined data set for children ages 6–17 years in the United States.^30^ This study also examines the mediating role of family resilience, a new measurement featured in the 2016 and 2017 NSCH, and the moderating influence of race/ethnicity and family poverty level (FPL) on the ACEs-flourishing link within an integrated moderated-mediation model. We hypothesized that family resilience mediates the relationship between ACEs and child flourishing and that these pathways vary by race/ethnicity and FPL.

## Study data and methods

### Population and data

Data from the 2016–2017 NSCH was used, a cross-sectional survey conducted annually by the US Census Bureau, which provides national and state-level data on the physical and mental health of non-institutionalized children, ages 0–17, from all 50 states in the United States and the District of Columbia. The NSCH was funded and directed by the Health Resources and Services Administration’s (HRSA) Maternal and Child Health Bureau (MCHB). Households with one or more children under 18 years old residing in the home were randomly surveyed. The survey was administered via web and paper-based instruments that were delivered by mail to either a parent or guardian of one child who was randomly selected as the subject of the survey. A total of 71,811 surveys were completed for the 2016 and 2017 NSCH combined data set. There was an overall weighted response rate of 40.7% for 2016 and 37.4% for 2017. The final analytic sample included 44,686 respondents reporting on children ages 6–17 years.^30^

### Key measures

Child-level household data provided demographic information (i.e., age, sex, race/ethnicity, and family poverty level), ACE summary counts, and questions about family resilience and child flourishing that were obtained via self-report from parents and guardians at the time of the interview. All variables used in this study have been documented previously, and their properties and coding are presented in publicly available NSCH variable codebooks.^30^

### Adverse childhood experiences (ACE) assessment

The nine ACE items measured by the NSCH included parent or guardian divorced or separated; parent or guardian died; parent or guardian served time in jail; witnessed or heard parents or adults slap, hit, kick, punch one another in the home; a victim of violence or witnessed violence in the neighborhood; lived with anyone who was mentally ill, suicidal, or severely depressed; lived with anyone who had a problem with alcohol or drugs; treated or judged unfairly because of his or her race or ethnic group; and economic hardship (e.g., hard to cover basics like food or housing). The NSCH did not include questions about emotional, physical, and sexual abuse or emotional and physical neglect that are found in the original ACE study questionnaire^6^ and state-by-state Behavioral Risk Factor Surveillance Survey (BRFSS).^31^ ACE survey items were coded as dichotomous variables that featured “yes or no” response options with the exception of economic hardship. The question, “How often has it been very hard to get by on your family’s income?”, was coded as a “yes” for “somewhat often” or “very often” responses and as a “no” for “rarely” or “never” responses. An ACE summary score was created by summing the nine dichotomous ACE variables and used as a continuous variable in the model analyses. Dichotomous ACE variables, including 0, 1, 2, 3, 4, 5, or ≥6 ACEs, were generated to determine prevalence.

### Child flourishing indicators

Child flourishing questions were formulated based on a review of positive health indicators by a Technical Expert Panel.^32^ A validated scale for measuring indicators of child flourishing does not exist. For ages 6–17, the three child flourishing questions asked, “How true are each of the following statements about this child”:  a) Shows interest and curiosity in learning new things,  b) Works to finish tasks he or she starts, and  c) Stays calm and in control when faced with a challenge. Response options were collapsed and dichotomized for each question to reflect that “definitely true” met flourishing criteria and “somewhat true” or “not true” indicated that the child did not meet flourishing criteria. The flourishing indicators represent three overlapping, yet distinct constructs of learning, resilience, and regulation. Each flourishing question was assessed individually as a separate outcome and coded as a binary variable (yes or no). The degree to which the individual constructs are impacted by cumulative ACEs can be best understood by retaining the specificity of the individual flourishing items.

### Family resilience mediator

Family resilience, a new measurement in the 2016–2017 NSCH data set, is a composite measure composed of four items including: “When your family faces problems, how often are you likely to do each of the following?” (a) Talk together about what to do, (b) Work together to solve our problems, (c) Know we have strengths to draw on, and (d) Stay hopeful even in difficult times. Response options were “none of the time”, “some of the time”, “most of the time”, or “all of the time”. A response of either “most of the time” or “all of the time” was required to meet each individual indicator. The latent variable of family resilience was reverse coded to show that larger values indicated higher levels of resilience.

### Race/ethnicity moderator

The moderating role of race/ethnicity was examined on the impact of cumulative ACEs on flourishing outcomes. The race and ethnicity distribution of the population was measured with the question, “What is this child’s race/ethnicity?”. Racial/ethnic status was coded as a trichotomous variable: 0 = “Hispanic”; 1 = “White, non-Hispanic”; 2 = “Black, non-Hispanic”. The category of “Other, Non-Hispanic”, children with more than one race category, were not included in this analysis due to insufficient numbers in the 2016–17 NSCH sample to provide reliable estimates.

### Family poverty level (FPL) moderator

The moderating role of FPL on the impact of cumulative ACEs on flourishing outcomes was examined. Household family poverty level was measured with the question, “What is the income level (federal poverty level, FPL) of the household that this child lives in?”. Response options derived from the Census Bureau included “0–99% FPL”; “100–199% FPL”; “200–399% FPL”; “400% FPL or more”. The first three categories were collapsed into one category and, subsequently, the measure was coded as a dichotomous variable: “below 400% FPL” and “400% FPL or above”. FPL uses an absolute threshold to measure poverty based on household income and size, whereas economic hardship is the perception of poverty and one’s ability to cover basic necessities. These variables measure different things and are not completely collinear.^33^

### Analytic strategy

**Fig. 1:**
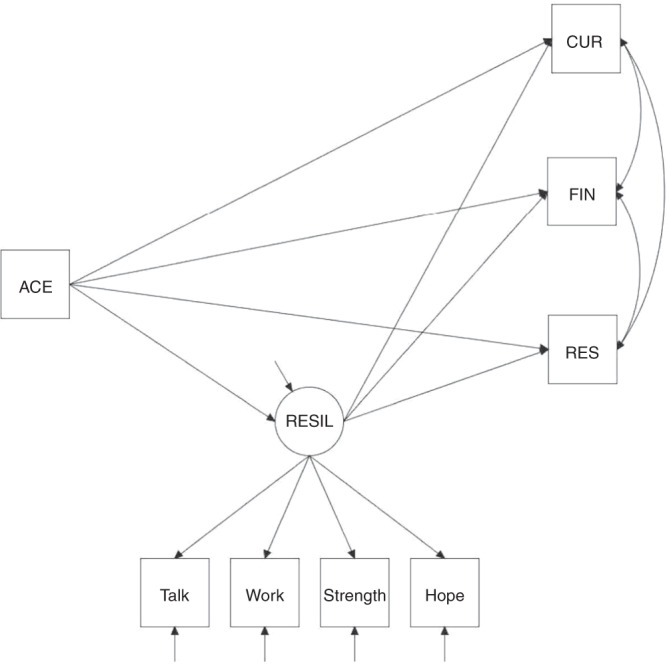
Moderated-mediation probit model of family resilience mediating ACEs impact on flourishing in children age 6–17 years. A probit model was used to examine a composite measure of family resilience (RESIL) as mediating changes in ACEs on specific indicators of flourishing for all moderating conditions (i.e., White versus Black vs Hispanic and above vs below family poverty level). The mediating structure was constructed on three binary outcome measures of flourishing: a) child shows interest and curiosity in learning new things (CUR), b) child works to finish tasks he or she starts (FIN), c) and child stays calm and in control when faced with a challenge (RES).

For this cross-sectional study, we used the 2016–17 NSCH data to examine associations between ACEs, flourishing, and family resilience, in addition to examining the mediating role of family resilience and moderating influences of race/ethnicity and FPL. Descriptive statistics were used to describe the characteristics of each variable in the sample using frequencies and proportions. The distribution of ACEs was cross-tabulated for race/ethnicity by gender and FPL for 0, 1, and ≥2 ACEs. Sample weighted correlations between the endogenous measures were obtained to evaluate the strength of the associations between flourishing items. Several correlation tests and sensitivity analyses were conducted to determine if any confounding was present between the ACE item that was asking about economic hardship and the FPL measure. Specifically, Cramer’s V was used to calculate the association between the ACE poverty item and the total ACE score, and a point-biserial correlation test was used to calculate the association between the ACE poverty item and FPL measure. In addition, a sensitivity analysis was performed to assess specifics involved with the overall latent measure. Survey sampling weights provided by the NSCH were used to account for gender, race, and ethnicity distributions for non-institutionalized children who live in the United States, as well as to adjust for nonresponses. A moderated-mediation probit model, shown in Fig. 1, was used to examine a composite measure of family resilience as mediating changes in ACEs on three specific indicators of flourishing for all moderating conditions.^34^ There is precedence for using structural equation modeling for simple mediational analysis. The inclusion of moderating conditions that reflect the disparities evident among diverse racial/ethnic groups and income levels require a more complex model. Since we were interested in multiple interactions within a system, we built a stacked model using categorical moderators to examine how those interactions would occur across the entire system. A stacked group approach using a probit link function with weighted least square parameter estimates was implemented to assess moderator effects. Mplus was utilized to estimate the direct, indirect and total effects of the model.^35^ This study also explored the adjustment of age and sex in the models, which were found to provide minimal effect and were subsequently dropped from the models.

## Results

### Measurement correlations

The sample weighted inter-item correlation matrix showed that the flourishing scale items were correlated (Supplementary Table 1). Additional modeling demonstrated that the direction of the effects was similar between the flourishing index and the multiple items that comprise child flourishing. Since  there were magnitudinal differences between flourishing indicators, disaggregating the effects of flourishing into individual components could yield further insight into how family resilience can help children realize positive outcomes in learning, regulation, and resilience across contexts of race/ethnicity and poverty.

The association between the ACE poverty item and the FPL measure was not strong (Cramer’s V = 0.3806). Similarly, the association between the ACE poverty item and the total ACE score was not strong (point-biserial correlation = −0.26). The ACE poverty item comprised approximately 7.47% of the total ACE score. A sensitivity analysis indicated that removal of the ACE poverty item from the total ACE score produced a nonconsequential impact on the estimates with minimal effect on the model results. These results provided justification for maintaining the ACE poverty item in the total ACE score and suggested that there should be little, if any, confounding of the ACE poverty item with the FPL moderator.

### Sample characteristics

Table 1 displays the sample characteristics for *n* = 44,686 children age 6–17 years. Nearly equal proportions of male and female children are represented by this sample, which includes Non-Hispanic White (80.13%), Black (7.07%), and Hispanic (12.8%) race/ethnicities. Slightly more than half of the children are from families living below the poverty level (56.5%).

**Table 1:** National Survey Children’s Health (NSCH) sample characteristics for children age 6–17 years, *n* = 44,686.

Variables	Frequency (*n*)	Proportion (%)
Demographics		
Sex		
Male	22,950	51.36
Female	21,736	48.64
Race/ethnicity		
Hispanic	5718	12.8
Black, non-Hispanic	3161	7.07
White, non-Hispanic	35,807	80.13
Federal poverty level (FPL)		
Below 400% FPL	25,237	56.48
400% FPL or above	19,449	43.52
Adverse childhood experiences (ACEs)		
Missing	597	1.34
0 ACEs	24,050	53.82
1 ACE	10,274	22.99
2 ACEs	4722	10.57
3 ACEs	2323	5.2
4 ACEs	1304	2.92
5 ACEs	774	1.73
6 or more ACEs	642	1.44
Flourishing (outcomes)		
Learning: interest and curiosity in learning new things		
Missing	236	0.53
Definitely true	37,291	83.45
Somewhat or not true	7159	16.02
Resilience: finish tasks		
Missing	750	1.68
Definitely true	28,557	63.91
Somewhat or not true	15,379	34.42
Regulation: calm and in control when faced with a challenge		
Missing	747	1.67
Definitely true	22,101	49.46
Somewhat or not true	21,838	48.87
Family resilience (mediator)		
Talk together about what to do when the family faces problems		
Missing	739	1.65
All of the time	18,192	40.71
Most of the time	19,799	44.31
Some of the time	5565	12.45
None of the time	391	0.87
Work together to solve the problem when the family faces problems		
Missing	844	1.89
All of the time	17,970	40.21
Most of the time	20,053	44.88
Some of the time	5450	12.2
None of the time	369	0.83
Know we have strengths to draw on when the family faces problems		
Missing	886	1.98
All of the time	21,367	47.82
Most of the time	17,633	39.46
Some of the time	4,347	9.73
None of the time	453	1.01
Stay hopeful even in difficult times when the family faces problems		
Missing	733	1.64
All of the time	21,729	48.63
Most of the time	19,257	43.09
Some of the time	2769	6.2
None of the time	198	0.44

Based on individuals with no missing values for sex variable.

### Prevalence and distribution of adverse childhood experiences (ACEs)

Just over half of respondents (53.82%) reported their children having no ACEs, whereas (45%) of children experienced at least one ACE from the 9 ACE items presented in the NSCH, which is the equivalent of 20,039 children. More than one-fifth of our sample, 22% (9,765) of children experienced ≥2 ACEs. Furthermore, 30.16% of children living below FPL experienced ≥2 ACEs compared to 11.07% of children living above FPL. Proportionally, 33.38% Black, 26.81% Hispanic, and 20.05% White children experienced ≥2 ACEs. Table 2 shows the distribution of ACEs by race/ethnicity with gender and FPL. Black male (34.61%) and female (32.07%) children had the highest proportion of ≥2 ACEs compared to Hispanic male (26.99%) and female (26.63%) children and White male (19.48%) and female (20.65%) children. Similarly, Black children living below FPL (38.61%) had the highest proportion of ≥2 ACEs compared to Hispanic (31.52%) and White (29.49%) children living below FPL.

**Table 2:** Crosstabulation of adverse childhood experiences by race/ethnicity with gender and family poverty level for children age 6–17 years, *n* = 44,686.

ACE score	Black, *n* (%)				Hispanic, *n* (%)				White, *n* (%)			
	Male	Female	Below FPL	Above FPL	Male	Female	Below FPL	Above FPL	Male	Female	Below FPL	Above FPL
0 ACEs	555 (34.13)	551 (35.90)	756 (31.72)	350 (49.23)	1322 (45.32)	1297 (46.30)	1662 (40.58)	957 (62.88)	10,503 (57.06)	9822 (56.45)	8081 (44.13)	12,244 (71.74)
1 ACE	473 (29.09)	460 (29.97)	707 (29.67)	226 (31.79)	762 (26.12)	704 (25.13)	1143 (27.91)	323 (21.22)	4101 (22.28)	3774 (21.69)	4829 (26.37)	3046 (17.85)
≥2 ACEs	563 (34.61)	492 (32.07)	920 (38.61)	135 (18.99)	787 (26.99)	746 (26.63)	1291 (31.52)	242 (15.90)	3585 (19.48)	3592 (20.65)	5400 (29.49)	1777 (10.41)
Missing	35 (2.15)	32 (2.08)	57 (2.34)	10 (1.39)	46 (1.58)	54 (1.93)	84 (2.01)	16 (1.04)	218 (1.18)	212 (1.22)	307 (1.65)	123 (0.72)

*ACE* adverse childhood experiences, *FPL* family poverty level.

### Proportion of child flourishing

The proportion of child flourishing outcomes reported by a parent or guardian as definitely true include showing interest and curiosity in learning (83.4%), working to finish tasks (63.9%), and staying calm and in control when faced with a challenge (49.5%).

### Proportion of family resilience

Regarding family resilience, families were likely to either most of the time or all of the time talking together about what to do (85.0%), work together to solve problems (85.1%), know that they have strengths to draw on (87.3%), and stay hopeful (91.7%) in difficult times.

### Direct, total, and indirect effects by race/ethnicity

Table 3a displays parameter estimates for direct, indirect, and total effects by race/ethnicity. As ACEs increased, flourishing indicators significantly decreased across race/ethnicity with the exception of interest and curiosity in learning for Black children, which decreased, however non-significantly. As family resilience increased, flourishing indicators significantly increased, and as ACEs increased, family resilience significantly decreased across race/ethnicity. Indirect effects demonstrated an average partial mediation of family resilience on the relationship between ACEs and flourishing for Black (37.5%) and Hispanic (30.6%) children.

**Table 3:** (a) Probit estimates with sampling adjusted weighting for the direct, total, and indirect effects of ACEs on flourishing outcomes by race/ethnicity of children ages 6–17 years. (b) Moderation effect parameter estimates differences with sampling adjusted weighting for direct, total, and indirect effects of ACEs on flourishing outcomes by race/ethnicity of children ages 6–17 years.

(a)	White		Black		Hispanic	
	Estimate (SE)	95% CI	Estimate (SE)	95% CI	Estimate (SE)	95% CI
Direct effects						
ACE → RESIL	−0.065 (0.004)*	[−0.073, −0.056]	−0.113 (0.015)*	[−0.142, −0.085]	−0.096 (0.011)*	[−0.117, −0.075]
RESIL → CUR	0.451 (0.027)*	[0.397, 0.504]	0.528 (0.071)*	[0.388, 0.668]	0.421 (0.066)*	[0.292, 0.550]
RESIL → FIN	0.453 (0.024)*	[0.405, 0.500]	0.379 (0.070)*	[0.242, 0.515]	0.423 (0.062)*	[0.302, 0.545]
RESIL → RES	0.477 (0.024)*	[0.431, 0.523]	0.414 (0.066)*	[0.285, 0.543]	0.376 (0.062)*	[0.255, 0.497]
ACE → CUR	−0.127 (0.011)*	[−0.148, −0.105]	−0.057 (0.030)	[−0.116, 0.002]	−0.066 (0.026)*	[−0.117, −0.015]
ACE → FIN	−0.159 (0.011)*	[−0.180, −0.138]	−0.097 (0.029)*	[−0.154, −0.040]	−0.107 (0.025)*	[−0.156, −0.058]
ACE → RES	−0.117 (0.011)*	[−0.139, −0.095]	−0.107 (0.028)*	[−0.162, −0.052]	−0.100 (0.027)*	[−0.152, −0.048]
Total effects						
ACE → CUR	−0.156 (0.011)*	[−0.178, −0.134]	−0.117 (0.029)*	[−0.175, −0.059]	−0.106 (0.026)*	[−0.157, −0.056]
ACE → FIN	−0.188 (0.011)*	[−0.209, −0.167]	−0.140 (0.029)*	[−0.197, −0.084]	−0.148 (0.025)*	[−0.196, −0.099]
ACE → RES	−0.148 (0.011)*	[−0.170, −0.126]	−0.154 (0.028)*	[−0.208, −0.100]	−0.136 (0.026)*	[−0.188, −0.084]
Indirect effects						
ACE → RESIL → CUR	−0.029 (0.003)* [18.59%]	[−0.034, −0.024]	−0.060 (0.010)* [51.28%]	[−0.080, −0.040]	−0.040 (0.008)* [37.74%]	[−0.056, −0.025]
ACE → RESIL → FIN	−0.029 (0.002)* [15.43%]	[−0.034, −0.024]	−0.043 (0.009)* [30.71%]	[−0.060, −0.026]	−0.041 (0.008)* [27.70%]	[−0.055, −0.026]
ACE → RESIL → RES	−0.031 (0.002)* [20.95%]	[−0.036, −0.026]	−0.047 (0.009)* [30.52%]	[−0.064, −0.029]	−0.036 (0.007)* [26.47%]	[−0.050, −0.022]

(b)	Hispanic vs White Δ		Hispanic vs Black Δ		White vs Black Δ	
Direct effects						
ACE → RESIL	−0.032 (0.012)*	[−0.054, −0.009]	0.017 (0.018)	[−0.018, 0.053]	0.049 (0.015)*	[0.019, 0.079]
RESIL → CUR	−0.030 (0.071)	[−0.170, 0.110]	−0.108 (0.097)	[−0.298, 0.083]	−0.077 (0.076)	[−0.227, 0.072]
RESIL → FIN	−0.029 (0.067)	[−0.160, 0.101]	0.045 (0.093)	[−0.138, 0.228]	0.074 (0.074)	[−0.070, 0.219]
RESIL → RES	−0.101 (0.066)	[−0.230, 0.028]	−0.038 (0.090)	[−0.215, 0.139]	0.063 (0.070)	[−0.074, 0.200]
ACE → CUR	0.061 (0.028)*	[0.005, 0.116]	−0.009 (0.040)	[−0.087, 0.069]	−0.070 (0.032)*	[−0.132, −0.007]
ACE → FIN	0.052 (0.027)	[−0.001, 0.105]	−0.010 (0.038)	[−0.085, 0.065]	−0.062 (0.031)*	[−0.122, −0.001]
ACE → RES	0.017 (0.029)	[−0.039, 0.073]	0.007 (0.039)	[−0.069, 0.083]	−0.010 (0.030)	[−0.069, 0.049]
Total effects						
ACE → CUR	0.049 (0.028)	[−0.006, 0.104]	0.011 (0.039)	[−0.066, 0.087]	−0.039 (0.031)	[−0.100, 0.023]
ACE → FIN	0.040 (0.027)	[−0.012, 0.093]	−0.007 (0.038)	[−0.082, 0.067]	−0.048 (0.031)	[−0.108, 0.012]
ACE → RES	0.012 (0.029)	[−0.045, 0.068]	0.018 (0.038)	[−0.057, 0.093]	0.006 (0.030)	[−0.052, 0.064]
Indirect effects						
ACE → RESIL → CUR	−0.011 (0.008)	[−0.028, 0.005]	0.019 (0.013)	[−0.006, 0.045]	0.031 (0.011)*	[0.010, 0.051]
ACE → RESIL → FIN	−0.011 (0.008)	[−0.027, 0.004]	0.002 (0.012)	[−0.021, 0.025]	0.014 (0.009)	[−0.004, 0.032]
ACE → RESIL → RES	−0.005 (0.008)	[−0.020, 0.010]	0.011 (0.011)	[−0.012, 0.033]	0.016 (0.009)	[−0.002, 0.034]

The latent variable of family resilience was reverse coded to show that larger values indicated more flourishing (*p < 0.05). Percentages in brackets are the proportion of the total effect explained by family resilience with 20% or more indicating partial mediation.

*ACEs* adverse childhood experiences, *CI* confidence interval, *RESIL* family resilience, *CUR* flourishing outcome of interest and curiosity in learning new things, *FIN* flourishing outcome of finishing tasks, *RES* flourishing outcome of staying calm and in control when faced with a challenge.

### Moderation effects by race/ethnicity

Table 3b displays the moderation effect parameter estimate differences for direct, indirect, and total effects across race/ethnicity groups. As ACEs increased, Black and Hispanic children experienced a steeper decline in family resilience compared to White children. However, White children experienced significantly greater decrements in flourishing compared to Black and Hispanic children as ACEs increased. For example, specific flourishing outcomes showed that White children experienced a steeper decline in interest and curiosity in learning compared to Black and Hispanic children. White children also showed a decline in working to finish tasks compared to Black children. The moderated-mediation effect of family resilience on the relationship between ACEs and curiosity in learning was statistically significantly greater for Black than for White children.

### Direct, total, and indirect effects by FPL

Table 4 displays parameter estimates for direct, indirect, and total effects by FPL, including moderation effect differences across levels of FPL. All direct, indirect, and total effect parameter estimates were statistically significant. As ACEs increased, flourishing and family resilience significantly decreased across FPL. As family resilience increased, flourishing indicators significantly increased across FPL. Indirect effects demonstrated an average partial mediation of family resilience on the relationship between ACEs and flourishing for children who were living below (27.7%) and above (21.0%) FPL.

**Table 4:** Probit estimates and moderation effect parameter estimate differences with sampling adjusted weighting for direct, total, and indirect effects of ACEs on flourishing outcomes by FPL of children age 6–17 years.

	Below 400% FPL		Above 400% FPL		Below FPL vs above FPL Δ	
	Estimate (SE)	95% CI	Estimate (SE)	95% CI	Estimate (SE)	95% CI
Direct effects						
ACE → RESIL	−0.087 (0.006)*	[−0.098, −0.075]	−0.073 (0.008)*	[−0.089, −0.057]	−0.014 (0.010)	[−0.033, 0.006]
RESIL → CUR	0.464 (0.036)*	[0.393, 0.535]	0.432 (0.041)*	[0.351, 0.513]	0.032 (0.055)	[−0.075, 0.140]
RESIL → FIN	0.428 (0.034)*	[0.362, 0.493]	0.449 (0.037)*	[0.377, 0.520]	−0.021 (0.050)	[−0.118, 0.076]
RESIL → RES	0.418 (0.033)*	[0.352, 0.483]	0.513 (0.034)*	[0.446, 0.579]	−0.095 (0.048)*	[−0.188, −0.001]
ACE → CUR	−0.083 (0.013)*	[−0.109, −0.058]	−0.117 (0.022)*	[−0.160, −0.074]	0.034 (0.026)	[−0.016, 0.084]
ACE → FIN	−0.122 (0.012)*	[−0.145, −0.098]	−0.141 (0.022)*	[−0.184, −0.099]	0.019 (0.025)	[−0.029, 0.068]
ACE → RES	−0.096 (0.013)*	[−0.121, −0.072]	−0.123 (0.024)*	[−0.169, −0.077]	0.027 (0.027)	[−0.026, 0.079]
Total effects						
ACE → CUR	−0.123 (0.013)*	[−0.149, −0.098]	−0.149 (0.022)*	[−0.192, −0.105]	0.025 (0.026)	[−0.025, 0.075]
ACE → FIN	−0.159 (0.012)*	[−0.182, −0.135]	−0.174 (0.022)*	[−0.216, −0.131]	0.015 (0.025)	[−0.033, 0.064]
ACE → RES	−0.132 (0.012)*	[−0.157, −0.108]	−0.160 (0.024)*	[−0.207, −0.113]	0.028 (0.027)	[−0.025, 0.081]
Indirect effects						
ACE → RESIL → CUR	−0.040 (0.004)* [32.52%]	[−0.048, −0.032]	−0.031 (0.004)* [20.81%]	[−0.040, −0.023]	−0.009 (0.006)	[−0.020, 0.003]
ACE → RESIL → FIN	−0.037 (0.004)* [23.27%]	[−0.044, −0.030]	−0.033 (0.004)* [18.97%]	[−0.041, −0.024]	−0.004 (0.006)	[−0.016, 0.007]
ACE → RESIL → RES	−0.036 (0.004)* [27.27%]	[−0.044, −0.029]	−0.037 (0.005)* [23.13%]	[−0.046, −0.028]	0.001 (0.006)	[−0.010, 0.013]

*p < 0.05.

### Moderation effects by FPL

 As ACEs increased, flourishing decreased slightly more quickly for above versus below FPL children, however this finding was non-significant. Statistically significant moderation effect differences for direct effect pathways demonstrated that compared to below FPL children, above FPL children were more likely to improve in their ability to stay calm and in control as family resilience increased. There were no statistically significant moderated-mediation effects of family resilience on the relationship between ACEs and flourishing in FPL groups.

## Discussion

The goal of this study was to examine the mediating effect of family resilience on the relationship between ACEs and child flourishing across varying race/ethnicity and FPL groups in a national sample of children ages 6–17 years. This study contributes to the existing literature by enhancing understanding of ACEs impact on child flourishing indicators of learning, resilience, and regulation and the protective familial factors and processes that can intervene on the ACEs-flourishing association, including the potential disparities inherent in these relationships across racial/ethnic and income groups.

Data indicate that the main variables were related in the expected directions. As adversity increased, families were less likely to demonstrate qualities of resilience and children were less likely to flourish across race/ethnicity and FPL. Although the association between ACEs and child flourishing may be best explained through several mediating variables, the partial mediating results highlight that family resilience is useful in understanding how ACEs are related to child flourishing. For example, White families were more likely to maintain family resilience in the face of adversity, although their children functioned more poorly at high-risk levels relative to Black and Hispanic children. Although family resilience reduced the negative impact of ACEs on flourishing in both races, there was a greater reduction for Black than White children. A more consistent income gradient for cumulative ACEs influence on flourishing by FPL could suggest that higher income is not necessarily a protective factor for ACEs impact on flourishing.

### Prevalence and distribution of adverse childhood experiences

Concordant with previously reported NSCH data,^15,33^ nearly half of U.S. children age 6–17 years nationally and in most states have experienced at least one ACE. Our findings show that ACEs are widespread, yet ACEs prevalence persists in being the highest among Black and Hispanic children and low-income families. Although race/ethnicity and income are not psychosocial adversities in and of themselves, they can confer the risk of exposure to ACEs and other traumas. Numerous social and health inequities create a concentrated disadvantage among Black and Hispanic children and those living below FPL, which leave them disproportionately vulnerable. It is highly likely that the actual adversities faced by U.S. children are underestimated by the NSCH ACEs measure, which excludes questions about abuse and neglect.

### Direct effects of ACEs, flourishing, and family resilience

As ACEs increased, the estimated probability of all indicators of flourishing outcomes and family resilience decreased across race/ethnicity and FPL, reinforcing the notion that ACEs are consequential for all children regardless of their background. Direct effect results in this study also indicated that family resilience increased child flourishing. As a result, increasing family resilience in the face of ACEs may also increase child flourishing. This hypothesis is further supported by reported findings from the 2011–12 NSCH data that showed children with two or more ACEs who were described as resilient were significantly more engaged in school and better able to maintain calm and control.^15,36^ In the 2016–2017 NSCH data, 50% of children did not meet flourishing criteria for staying calm and in control when faced with a challenge and, therefore, increasing self-regulation for children with ACEs is a priority area for focusing resilience efforts. We can gain a more nuanced understanding of enhancing and tailoring prevention and intervention efforts to effectively address ACEs impact on learning, resilience, and regulation for diverse racial/ethnic and family poverty level groups by analyzing the distinct pathways of flourishing indicators.

### Mediating influences of family resilience on ACEs and flourishing

Family resilience was found to be a partial mediator, suggesting that resilience is a multi-factorial process and that there are additional potential mediating influences that can also explain the association between ACEs and child flourishing outcomes. Previous studies using NSCH data have identified a number of partially mediating factors of adversity and flourishing such as residing in a safe neighborhood, attending a safe school, and parental monitoring of friends and activities.^20^ Several studies among resilient Black and Hispanic children point to strong values of family cohesion and cooperation and greater intergenerational interdependence and social supports outside the immediate family as determinants of resilience that can contribute to lessening the impact of adversity.^37^ A combination of child, family, and community-level factors can serve as protective processes in promoting resilience for vulnerable children.^38^

### Moderating effects by race/ethnicity and FPL

Moderator findings underscore the disproportionality of cumulative adversities affecting Black and Hispanic children and families and those living in socioeconomically disadvantaged communities. The partial mediating effect of family resilience in the relation between ACEs and child flourishing varied across different race/ethnicity and FPL groups. Findings revealed that family resilience may be more difficult to muster for Black and Hispanic children, although children in these families tend to function slightly better at high levels of adversity relative to their White counterparts. A similar trend was observed between above and below FPL, however, there was less variance between groups indicating that ACEs are distributed more evenly across income levels and not just concentrated in socioeconomically disadvantaged families. One possible explanation may be that previous exposure to some or moderate amounts of stress can result in a steeling or hardening effect that can promote resilience to later stress and may operate at high levels of ACEs for some people and groups of people.^39^ These findings warrant further attention as they are consistent with prior research that emphasizes the differential effects of ACEs by race/ethnicity and income in U.S. children.^25,28^

### Limitations

Several limitations should be noted. In the absence of longitudinal population-based data that includes ACEs exposures, the indirect pathways were analyzed using cross-sectional data. A correlational study design. precludes interpretations of causality and insights based on temporal precedence. Although the NSCH ACEs measure excluded indicators of child abuse and neglect, research suggests that the assessed ACE items often co-occur with abuse and neglect.^7^ Consequently, results derived from measuring cumulative risk likely include children with such experiences, though not all. Still, understanding the actual distribution of ACEs is key to developing effective interventions and prevention efforts. Moreover, self-report data by a parent or guardian may be subject to respondent bias due to hesitancy of sharing openly about their family and the associated stigma, which in turn can contribute to underestimates of adversity exposure and overestimates of family resilience. Finally, this data excluded children who are doucumented in research as having high levels of disadvantage and ACEs, such as children who have been institutionalized and are without an address, in addition to under-sampling racial and ethnic minorities. The absence of these children limits our understanding of the impact of ACEs on vulnerable and marginalized groups. Overall, these limitations point to the need to develop more sensitive and accurate ways to measure exposure to ACEs that include household and community-level stressors and their effects across the life course.

### Future directions

Despite these limitations, findings from this study offer preliminary evidence that can guide future research. Examining an ACEs dose–response effect within the context of risk and protective factors and processes using national, population-based data can inform a public health response that can result in a larger impact for future prevention efforts. Studies based on national samples provide credible data to support the role of federal agencies in directing financial support and resources to ACEs prevention and intervention efforts. Supportive policies for early intervention can promote long-term psychological health and well-being. Considering this study’s findings along with other literature, it is important that future research explore an interrelated system of an individual, family, social, and other community-level protective factors that may play a role in lessening ACEs impact on child flourishing; in addition to understanding how race/ethnicity and FPL, in combination, influence outcomes and effects particularly in longitudinal studies.

ACEs are a result of modifiable and preventable disparities,^29^ and are identifiable root causes of medical problems.^24^ Sociocultural factors such as race/ethnicity and socioeconomic status are among the most influential determinants of health and disease in the population.^40^ The inequities intrinsic to these factors play a stronger role in influencing health and determining outcomes than either health care or individual health behaviors.^41^ Since ACEs are distributed widely across race/ethnicity and FPL and not just concentrated in children from Black and Hispanic and socioeconomically disadvantaged families, both universal prevention and targeted intervention efforts are needed.^25,28^ ACEs conversations should be routinely incorporated into well-child visits to provide foundational knowledge of toxic stress and health and to share resilience-promoting resources with children and parents as early as possible.^42^ Addressing ACEs within a social determinant of health and trauma framework using a two-generational approach can improve the ability to reduce health and achievement inequities while strengthening resilience for children and families facing many risks.^43^ ACEs education and resources are valuable to children and families whether or not a child has been exposed to ACEs, since learning how to recognize and manage stress and learn resilience is fundamental to development and child flourishing.

## Conclusion

ACEs are detrimentally impactful regardless of one’s status, and high ACEs, in particular, are nearly insurmountable across race/ethnicity and FPL. At the same time, it is important to recognize the vulnerability of racial/ethnic minorities and low-income family resilience to ACEs exposures and effects. The prevention of ACEs should be the number one charge of public policy, programs, and health care. Among sectors providing services to children and families who have been exposed to ACEs, we can increase our predictive powers of adversity-related risk and resilience by assessing protective and social factors in addition to the dose–response relationships to various negative outcomes. ACEs resources should be made available in every setting that serves the needs and touches the lives of children and families.

## Supplementary information

Supplement Table 1
