# Supplementary material for: Influence of race/ethnicity and income on the link between adverse childhood experiences and child flourishing
Source: Pediatr Res. 2020 Oct 12;89(7):1861–9. doi: 10.1038/s41390-020-01188-6 (PMC8249234; doi:10.1038/s41390-020-01188-6)
Supplement: Supplementary file 1 — Supplement Table 1 [file 41390_2020_1188_MOESM1_ESM.docx]

Supplement Table 1. Sample weighted correlation matrix

|  | Talk | Work | Strength | Hope | ACE Count | Learning | Regulation | Resilience |
| --- | --- | --- | --- | --- | --- | --- | --- | --- |
| **Family Resilience:** |  |  |  |  |  |  |  |  |
| Talk |  |  |  |  |  |  |  |  |
| Work | 0.883 |  |  |  |  |  |  |  |
| Strength | 0.778 | 0.836 |  |  |  |  |  |  |
| Hope | 0.66 | 0.71 | 0.782 |  |  |  |  |  |
| **ACE Count** | -0.151 | -0.177 | -0.177 | -0.156 |  |  |  |  |
| **Flourishing Items:** |  |  |  |  |  |  |  |  |
| Learning | 0.265 | 0.285 | 0.313 | 0.276 | -0.194 |  |  |  |
| Regulation | 0.255 | 0.275 | 0.282 | 0.264 | -0.237 | 0.679 |  |  |
| Resilience | 0.233 | 0.28 | 0.277 | 0.28 | -0.2 | 0.524 | 0.642 |  |
